# Covert Contraceptive Use amongst the urban poor in Accra, Ghana: experiences of health providers

**DOI:** 10.1186/s12978-022-01516-5

**Published:** 2022-11-04

**Authors:** Mawuli Kushitor, Elizabeth G. Henry, Akua Danquah Obeng-Dwamena, Martin Wiredu Agyekum, Caesar Agula, Theophilus Toprah, Iqbal Shah, Ayaga A. Bawah

**Affiliations:** 1 Fred Newon Binka School of Public Health, University of Health and Allied Sciences, Ho, Ghana; 2 Regional Institute for Population Studies (RIPS), University of Ghana, Accra, Ghana; 3 Department of Global Health and Population, Harvard T.H. Chan School of Public Health, Boston, USA

**Keywords:** Covert Contraceptive Use (CCU), Health providers, Health facilities, Provider support, Ghana

## Abstract

**Background:**

An estimated one-third of women in Ghana use contraceptives without the knowledge of their partners, a phenomenon known as Covert Contraceptive Use (CCU). Most research on CCU to date has focused on individual women to the neglect of the role of health system. This study explores CCU in urban poor communities of Accra, Ghana, from the experiences and perspectives of health providers.

**Methods:**

Qualitative in-depth interviews were conducted with health care providers in both the public and private sectors at multiple levels, from the community clinic to the tertiary hospital, to gain insights into the strategies women use and the ways in which the health system supports the practice of CCU.

**Results:**

Five major thematic areas emerged: use of easily concealed-methods, discrete-access-and-information-keeping, time-of-day, non-verbal-communication and use of relationships. The study further revealed that fear, mistrust, shyness, myths, and misperceptions regarding contraceptives explain CCU among women in the communities that the providers serve.

**Conclusion:**

Importantly, disclosure of methods used by providers without women’s consent could potentially lead to violent outcomes for both women and the providers. Our results highlight the pivotal role that providers play in confidentially supporting women’s choices regarding the use of contraceptives.

## Plain Language summary

Sometimes women use contraceptives without the knowledge of their sexual partners. That is to say, they hide their contraceptive use. Many studies have explored why women hide their contraceptive use. Reasons include the desire of men to have absolute control of their women’s bodies including their sexuality and fertility regulation. This occurs in cultures where women do not have as much power as men. In Ghana, as much as one-in-three women hide their contraceptive use from their partners. This study explores the ways in which health facilities assist women to hide their contraceptives use by talking to leaders of units responsible for providing contraceptives in the hospitals. First, people prefer contraceptives like injectables that are not visible on the bodies of those using them. Second, some hospitals have modified their space to provide ample privacy and security for women. Third, some women visit the facilities at odd hours, such as very early in the morning, very late at night and at other favorable times. Sometimes nurses arrange to meet women outside the facility. Lastly, nurses create avenues through various personal relationships. For example, leaving their contacts in market spaces and arranging meeting places with market queens.

## Background

Globally, many women use contraceptives without the knowledge of their partners [1, 2], a phenomenon described as Covert Contraceptive Use (CCU). The practice of CCU seems to vary greatly by region and among population sub-groups. A little over 12% has been reported in Kenya while over 30% has been reported in Ghana [1, 3].

The phenomenon of CCU underscores major differences in reproductive expectations of men and women. CCU also highlights the power imbalances between men and women in marital unions [4]. While men may assert themselves in marital/sexual unions, women may subtly express control of their bodies and the livelihoods by choosing contraceptives covertly. Research further suggests that CCU is indicative of power imbalances within the context of unions [5]. In many instances, women exercise little autonomy over their own sexuality and reproductive health [6]. Misconceptions on the proper use and intent of contraception may create mistrust amongst couples and prevent the discussion about and use of contraception especially in patriarchal societies. So men have, in the past, sometimes associated contraceptive use with spousal infidelity, and this has been seen in more recent literature from neighboring Nigeria [7, 8]. Additional research shows that women may not discuss their contraceptive decisions with their partners [9, 10]. The nature of the relationships between partners has been suggested as the main driver of CCU [10]. In these contexts, where women face barriers to using contraceptives due to their interpersonal relationships, women may look for innovative ways to hide their use of contraceptives [11, 12].

In Ghana, the prevalence of CCU has previously been documented at 34% among women attending a reproductive health clinic. Research suggests that women expected, and were often provided support from health providers. Relationship quality, trust, and the inability of a woman to exercise autonomy over her own body have been cited as reasons for CCU [12, 13]. The health care system has been a major facilitator of CCU, yet, very little is known about how the health system within this context may initiate and support covert use of contraceptives. Ghana operates a decentralized health system at three levels of organization. The Community-Based Health Planning and Services (CHPS) operates at the periphery of this complex system. CHPS are often located within small community settings. Health centers and district hospitals are levels above the community. District and tertiary level hospitals provide specialized and more advanced care to larger population groups. Little is known about the modes and strategies deployed to conceal contraceptive use in Ghana across all levels of the health system, and most studies conducted in SSA only focus on individual level factors associated with CCU neglecting the contribution of health care providers who provide the mechanism and sometimes the resources for successful concealment of contraceptives [3].

It is unclear to what degree the need for CCU is not being met and may thus be hindering progress in further reducing unmet needs for family planning overall. In addition, although CCU is widely reported in SSA, a comprehensive understanding of how contraceptives are delivered covertly is limited. A nuanced understanding of CCU may help improve the conduct of family planning programs in similar contexts in other developing countries. The objective of our study was to explore CCU from the perspective of providers within the health care system in Accra, Ghana. We hypothesized that providers’ perspectives on women’s CCU would provide a unique explanation for how CCU operates in the Ghanaian context, and explore factors that would be otherwise unrecognized.

## Methodology

### Study design and site

This study uses qualitative data collected as part of a cross-sectional study conducted by the Harvard T.H. Chan School of Public Health and the University of Ghana to assess the current practices and perceptions of providers regarding the provision of sexual and reproductive health services in four distinct neighborhoods of coastal Accra. Qualitative in-depth interviews were conducted with health care providers providing services at both public and private health facilities throughout the study communities. Structured interview guides were developed by the team of researchers to explore the experiences of health providers and their observations of women’s practice of CCU. The study is also informed by the literature on CCU.

### Study setting

The four communities in our study, Osu, La, Teshie and Nungua, are indigenous Ga communities that are characterized by multiple generations of families living together in large family houses. At a higher level, these communities are structured around informal chieftaincy institutions. Over the past few decades, these communities have experienced massive population growth due to rural urban migration. The already crowded indigenous communities have been at the receiving end of large movements of people from the hinterlands. According to the latest population and housing census conducted by the Ghana Statistical Service, the four areas put together have a total population of 700,000 people [14]. Slums have developed from rapid population growth without the corresponding infrastructural development. Typical of slums in Africa, these communities are characterized by lack of infrastructure, overcrowding, poor sanitation, high fertility, joblessness, poor quality housing, social disorder and social mistrust [15–17]. Besides these structural challenges, there is evidence to suggest that risky sexual behavior is more associated with living in slum areas. Typically, those living in slum areas have an earlier sexual debut, that is become sexually active earlier, are associated with lower use of modern contraceptives and are more likely to have multiple sexual partners [17]. Further, within the contexts of slums in Accra, school dropout rates are higher and most people are employed largely within the informal sector. The fishing industry dominates economic activities of all four study communities. Women are typically involved in the sale of street foods and in petty trading [18]. The communities have very similar profiles, although Nungua is larger, and together they form a long continuous stretch across the coast of Accra.

### Participant selection and fieldwork

This study applied a two-stage process to select the study participants. The first stage involved a thorough and complete listing of all health facilities in the four study communities. A total of 47 facilities were identified, out of which 20 facilities were dropped because they did not provide family planning services. With the help of the directorate of the Ghana Health Service, 13 facilities out of the remaining 27 were identified and selected for the study. The selection of these facilities was based on keeping a comprehensive geographical distribution across all four communities, the level of the health facility, types of family planning (commodities) provision and the level of contraceptive use that was reported in a separate facility assessment study. For all these facilities, health professionals who were in-charge of running the family planning units were invited for an interview. Ethics approval and consent to participate was obtained from all respondents and audio recordings of all interviews were made after obtaining the consent. All interviews were transcribed verbatim.

### Analysis

A thematic content analytical approach was adopted. Themes and their frequency are important for interpretation. As a result, a grid was developed to map out the occurrence of themes by respondents. The coding and analysis were facilitated by the qualitative software package, Atlas.ti. version 8. The analytical framework was based on a coding frame with two main subsections: The first coding section was developed from a system of pre-existing codes derived from family planning qualitative research literature in Africa and beyond [3, 7, 19–22].

The second section of the coding used inductive codes and focused on culturally specific ideas related to family planning not yet captured by the literature. A team of five researchers from the University of Ghana coded the data collectively. A code was defined as a basic unit of an idea. There were two levels of coding. Initially, the team picked out every unit of idea within the transcripts. The team had extensive engagements on each transcript to ensure that appropriate coding and labelling was done. The second level of coding involved grouping together similar codes or themes into organizing themes. Organizing themes were mainly driven by sub-sections of the interview guide. The interpretation of the results was facilitated by multiple discussions focused on consensus, conflicts and absences.

## Results

### Characteristics of study participants

A total of 13 health care providers were interviewed. Respondents varied by years of experience and rank. Two Deputy Directors of Nursing Services (DDNS) participated in the study. The DDNS is almost the highest professional attainment in nursing services in Ghana. Both of these were based at the tertiary and district facilities. There were six midwives from the district and public facilities and three community health nurses for the lower-level CHPS compounds. The experiences of these interviewees ranged from 2 years to almost 40 years of nursing care in Ghana. Two of the respondents were interviewed in their last week of work before retirement. The different professional and experiential perspectives provided a rich diversity of opinions on family planning. There was no refusal.

### How health facilities support CCU

Five main thematic areas emerged which describe strategies and adaptations in regular service delivery that have been informally employed at the health facility level to support clients in their practice of CCU. The first and most dominant was the health system provision of choice of easily concealable methods followed by discrete access and information keeping, time of day, the use of non-verbal cues and strategies that are based on direct relationships between women and health care providers. For most CHPS compounds, health providers were often expected to live close to the health care facilities. Health care providers therefore had many opportunities to directly engage with community members through several avenues such as regular outreach programs and the provision of walk-in-services. In addition, community health nurses were specifically trained to provide health care services to the community members. Over time, health providers developed relationships with community members through these multiple engagements. Further, almost all participants were directly in charge of running the family planning units. They were therefore well informed and able to respond sufficiently to the research instruments.

#### Easily concealed methods: “The one that people cannot see”

The results revealed that while considerations regarding side effects of FP were important in determining the choice of contraceptive, providers in some cases observed women preferentially selecting methods that could be used discretely. For this reason, providers believed that the 1-month and 3-month injectables were highly patronized because most women felt sure their partners would not be able to detect their use.

*“They said they like the injectable because when the person injects it you will not see it on her. If you inject her on the shoulder or on the buttocks and she is going, you cannot identify anything but with the other methods somebody can see. Unlike the Jadelle [implant] that you put here, the person can point and see that there is something there. The condom in usage the person will see, the pill too you will see so they don’t want the one that will show. They prefer the one that people cannot see.”*

*-Family Planning nurse-in-charge, Private clinic*

Some health workers noted women’s preferences for IUDs and other longer-term methods. These methods were also perceived to be popular amongst some women because they could also be concealed and had the added benefit of providing coverage for women for a longer-term, whereas injectables require returning to the clinic on a monthly basis or every 3 months.

*“Some ask if their husband won’t feel the thing on the arm. Some prefer that than the three months.” ….*

*-Community Health Nurse, CHPS Compound*

#### Discrete access and information keeping

Over time, health facilities have adapted to the needs of their clients regarding discrete use of FP by making changes within the physical space of the clinic. For example, all facilities included in the study had compartments for keeping hospital cards of women who did not wish to take their family planning records home.

*“Some people come and they say they are not ready for another [baby] but their partner is insisting that they should give birth. So with such people they come and do it and keep their cards with us….”*

*-Officer-in-charge of Family Planning Unit, Public Polyclinic*

Generally, health facilities isolate FP related activities from general outpatient activities to create a sense of privacy mainly because of the weight of stigma and misperception associated with FP at the community level.

*“One of the counselling rooms has been isolated for adolescents only. That’s where we are now, that’s how it has been labeled as adolescent’s corner. So, we have four counseling rooms and two procedure rooms” ….. -Family Planning Nurse, General Hospital.*

Some facilities had created isolated spaces outside the view of the general public where FP services could be provided in secret should the service be required. The strategy of one small facility is worthy of mention. They had created a secret door behind the facility known to only a few people where a nurse waited for patients who had been specially referred there. The referrals were made by nurses during community rounds or when a special FP need was required outside the view of people visiting the hospital.

*“We have the back door here so that people will not see them. So that those passing by will not see them, those coming for CWC [Child Welfare Clinics] will not see them. They use the back door and we do the counselling and based on the counselling, most of them agree to do it. Upon the counselling then they will make their informed choice. A lot know about family planning but because of the perception… so we clarify the perception and they agree to do it” ……….*

*-Community Health Nurse, CHPS Compound*

#### Time of the day

This thematic area was associated with only clients who obtained services from community health nurses. Respondents observed that community members would request to see the nurse outside of working hours for FP services. Times noted included late night, very early hours of the morning and sometimes after closing hours when clients believed that the health facility would be empty of people. Time was an important element, given that the location of CHPS in the community could easily reveal those women who were attending the clinic.

*“.. so she told me that later the people were coming in the night… the people were coming to the clinic at night when she was asleep or preparing her meal…”*

*-District Health Nurse (DDNS), District Health Management Team (DHMT, Lead)*

#### Non-verbal cues for requesting service

Teenagers had developed interesting gestures to attract the attention of nurses outside of the clinics when they wanted FP services. Sometimes they would walk around the facility hoping to attract the attention of the nurse on duty. The case of teenagers being observed by community members near clinics was more precarious as they were not expected to be seen at a hospital/clinic except for when it was obvious they were sick. Some nurses were able to pick some of these non-verbal cues and support the teenagers’ desire to obtain FP, as noted by the quote below.

*“[inaudible]…spy to see what the person is doing. So at times I chase them to talk to them. So I tell them not to worry because their mothers will go. So, I give them days that we don’t have the clinic and when they are coming they should use the back door. Or when they are ready I meet them at their house and I talk to them about … even though I have finished talking to them and they have other questions they call me and at times we go to their houses to provide the service…”*

*-Officer-in-charge CHPS compound*

In certain homes, parents go to work during the day and adolescents are left alone in the homes. Nurses may be aware of this opportunity and expressed that they have used this time and space to address reproductive health issues.

#### Use of relationships

Nurses mentioned visiting homes when they were sure a woman would be alone to receive FP services. They would leave their contact information such as mobile phone numbers on information boards in market places, community centres etc. Sometimes, the contacts of nurses were kept with safe community key informants such as market queens who have influence over large number of market women. The African informal market is a space essentially dominated by female traders, and the market queen is the traditionally designated head of women traders. She is directly responsible for all trading activities in the market and is considered the head of the women. Other safe contacts were queen mothers and members of the community health committees. Both the queen mothers and the market queens are a part of the social/traditional setting of most communities in Ghana and other African settings. They are traditionally charged with taking care of the welfare of women. Nurses would also use a network of women who had successfully and quietly used FP to get the word out to other women in the community who may need covert contraceptive services.

*“No, they come… [when] we go for home visit we get them and we tell them about family planning. At times we get groups of people and some of them feel shy or bad when they come out of that group to do the family planning. So we take their [phone] numbers when we go for the home visit” ….. Community Health Nurse, CHPS Compound*

As a last resort, nurses sometimes referred women to facilities in distant locations when the risk of disclosure was just too much.

*“They gave us a lot of names and contacts. Different hospitals, from Ashaiman to Ada and I have those numbers. Some are in Accra but they don’t want to do it in Accra, they want to go to other places because of the stigma”. ……… Community Health Nurse, CHPS Compound*

### Reasons for the practice of CCU

**Fig. 1:**
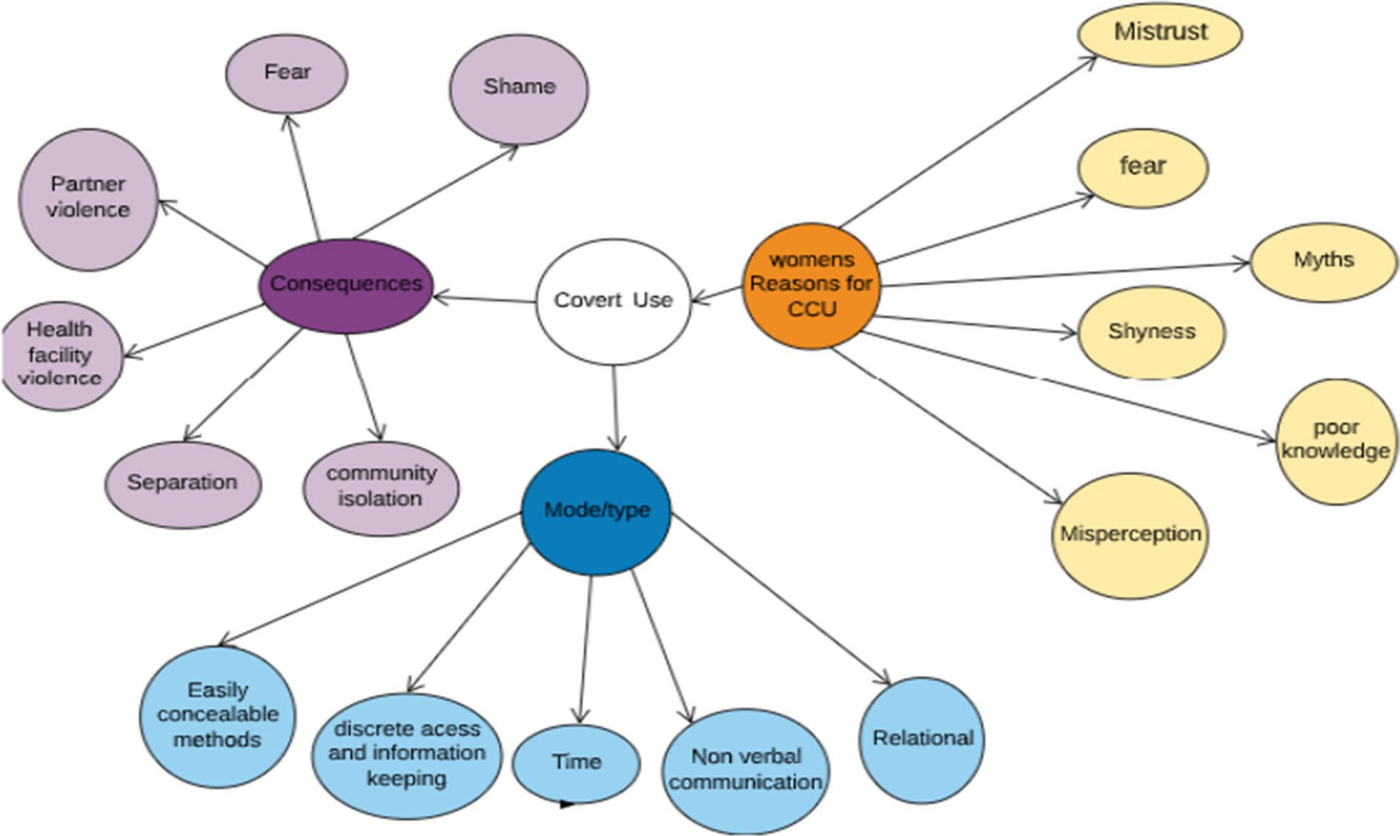
Type, reasons for and consequences of covert use in urban Ghana

Two major themes emerged to help explain the practice of CCU within these communities from the perspective of health providers. These were “Fear, Mistrust, Shame and Shyness” and then “Misperception and Myths Associated with FP Use”. Figure 1 illustrates the types, reasons and consequences associated with covert FP use.

#### Fear, mistrust, shame and shyness

Providers expressed observing visible changes in their female clients’ behaviours when their patients believed that concealment of their FP use was threatened.

*It is not easy because even if they come here and they meet someone they know, their mannerism and everything changes. Although there are some people who discuss when they meet here, others run away when they meet other people they know here. …. Midwife, General Hospital*

Fear associated with confrontation by partners is not the only driving force of covert behaviours, but sometimes “shyness” also has the same effect. This is typically associated with married couples who do not want to be seen using contraceptives. Within communities, the use of contraceptives has been associated with infidelity. This perception is true for both married couples and for those in other kinds of sexual relationships such as co-habitation.

*……. some of them feel shy that they are having sex. I am a Christian and I go to church and if I tell somebody about my reproductive issues they will think I am having sex [referring to marital infidelity]. So they will prefer they keep it to themselves or send it elsewhere…Midwife, Government Poly Clinic*

#### The use of disclosure against women who use FP

In terms of CCU, providers stated that women are not only concerned about their sexual partners but also the broader social community as well. Women were perceived as constantly on the lookout for potential threats. They would guard their use of FP sometimes from friends and the community at large. Disclosure can easily be deployed against the women during neighbourhood confrontations.

*Women, [we] talk a lot. So they will not like to bring out their [true] feeling [about contraceptive use] or tell friends about it. They can insult you with it one day [disclosure can be used against you]. So, it is easy for the men to come out rather than the women…Principal Community Health Nurse, Public clinic*

*So they study each other, if they know that the others or the friends or their peers are not using it they will not talk about it because their peers will go and talk about it [disclosure can be deployed against them]…. Deputy Director of Nursing Services, Tertiary Hospital*

##### Potential violence against women and health providers

For some facilities, there was a history of confrontation from men about their spousal contraceptive decisions. They came to the facility to warn health care workers against providing contraceptives to their partners. Others came to their facility to ask nurses to discontinue the family planning for their partners.

*Interviewer: yes, the few men who come here, what drives them to come for family planning?*

*Respondent: that is when they don’t want their wives’ to take the family planning*

*Interviewer: so they come here to tell you that you shouldn’t let their wife’s take the family planning?*

*Respondent: yes, some of them …….. Community Health Nurse, CHPS Compound*

#### Misperception and myths associated with FP use

Misconceptions were widely circulated through the communities and sometimes through social media. The quotes below illustrate some of the noted misconceptions and how the health services had attempted to deal with them.

*I have forgotten but there are a lot that you will hear and laugh. Some say when you do it, you will be punished by God because the Bible tells us to go and give birth and fill the earth so why do you want to prevent that. So when you do that it means you will be going to hell………… Deputy Director of Nursing Services, Municipal Health Directorate*

*It was easy but at first it was like the Muslims don’t do it because it is haram. But when we realized that they need permission from their spouses. There was one Alhaji who was our health promoter and he told us this. When we started it was not easy but now it is marvellous. Now there is no hiding even though some think that when they do it, they will be promiscuous meanwhile they are chasing other girls and they don’t want their partners to do it……… Deputy Director of Nursing Services, Municipal Health Directorate*

*yes, it was on a low previously but with WhatsApp [messenger]… sometimes they will send you a WhatsApp message condemning family planning. Especially the Community Health Officers, that when you take it you will get cancer. When we see it, we also forward it to ourselves and discuss it because it is fear and it is a concern. You will see the people withdrawing because they have heard it gives cancer and nobody would want to get cancer. That is the unsafe aspect of it………….. Deputy Director of Nursing Services, Municipal Health Directorate*

### Consequences of CCU

Health workers expressed their preference for CCU, especially when pregnancy could jeopardize the health of the woman. At the same time, providers also discussed their awareness of the social and cultural inhibitions against the use of FP services within the context of the community and that they had to consider this when providing advice to their clients. There were several consequences, some of the themes noted were accusations of infidelity, community isolation, fear, and in some cases violence against women and sometimes against health care providers by aggrieved partners.

#### The accusation of promiscuity and marital infidelity

For some reason, community members tended to associate the use of contraceptives with promiscuity for those not in recognised unions and marital infidelity in the case of those in recognised unions.

*Some also say that those who do it are promiscuous. …….. District Director, DDNS*

The strong perception of infidelity associated with contraceptive use leads to isolation, intimidation and sometimes weaponized against individuals using contraceptives within the context of the community.

*She was saying that the community members think that those who do family planning are promiscuous in a way because some people have a quarrel and one party used family planning to insult the other and the one who does family planning was deflated. When the other person said that she became confused and she couldn’t [continue with the fight] …. District Director, DDNS*

#### The threat of violence

Women make contraceptive decisions in consultation with nurses. The risk of disclosure is not only on women but sometimes on providers as well.

*She wanted a long-term implant fixed and she insisted I do it for her but from the conversation I had with the guy on the phone, it was with threats. I didn’t know what to do as a health worker because the client wanted it and she was ready to bear the consequences at home but I was also not comfortable to do it for her because I perceived that if she doesn’t come with her belongings after the guy has thrown her out, she might come back to the facility with a swollen face. So, I consulted my senior colleague on this and we agreed to give her the injectables. So, we have her attendance book. We only tell her the date and she comes for the injection and goes back home …. Midwife, General Hospital*

#### Poor attitudes of some health workers

Besides partners and community nurses, the conduct of some health providers provided an extra layer of difficulty in accessing and continually using contraceptives. The quote below illustrates how some health providers frustrate teenagers.

*“At that age especially those in school, they feel when they come here, we might insult them and ask them what they want family planning services for. Especially when they are coming they will be thinking about what people will be thinking about them. That makes a lot of them not come to us and they will be moving to the pharmacy to buy “Secure” [a brand of contraceptive pill popular in Ghana]. They think when they come the nurse will insult [them] and what the people around them will say so they will not come. Because she is [in] school and has not grown yet but she is coming in for family planning… personally I will not insult you or judge you but another colleague can do that……” Community Health Nurse CHPS Compound*

*“Yes, it happens a lot. We do get a lot of cases that the woman has delivered or has about six children or three or four and the woman does not feel strong to continue with the deliveries and the man refuses, the woman will come and have her family planning method done and the man will not be aware and it is the woman’s right. It is not compulsory that the woman should go and ask permission from the husband but we do encourage them to get their partners involved because of our culture but when the man says [no, you will not practice family planning]. Meanwhile you know at a certain age… the woman’s age will be circled, if the woman is from 34 or 35 going and with more than four children, the woman’s age and the number of children will be circled and the woman will belong to the at risk group. If the woman gets to that stage and she feels she can no longer make babies, -I am not strong so I am okay with the two or three that I have and the man refuses, the woman has her own right to practice family planning without the man’s concern… DDNS, Tertiary Public Facility*

While nurses were concerned about the conflicts associated with disclosure in some circumstances, they were more concerned about the implications of non-use of contraceptives resulting in pregnancy and potentially unsafe abortion. The crude and unsafe methods deployed to abort unwanted pregnancies are the real concern for health care providers.

*No one knows where they even get the drugs from. They only come back in a state that is not good and the doctors have to intervene. You should see the methods that some of them use. Some say they insert a stick into their private parts. Some of them take herbal drugs, others take cytotec whiles others insert it. They have so many methods. They run to the hospital when the labor starts and they can’t bear the pain; that’s when they run to the hospital……. Midwife, Quasi Government Hospital*

The risks associated with unmet need for contraceptives are not only associated with risky sexual behaviour for unmarried women, but also for those even in stable unions. Health workers note that some partners are difficult to manage and their behaviour often jeopardizes the health of their partners. In the quote below, the nurse explains the difficulty of engaging some male partners. In the face of such unyielding and potentially violent partners, CCU remains the only option. The partner even threatened to uncover covertly provided contraceptives.

*Yes! I had an incomplete abortion case where the lady had tried to terminate the pregnancy on her own. We completed the abortion for her. After we tried to counsel the person, when I counseled her, she asked me to speak to her boyfriend. So I explained to her that if the boy is not in agreement that she does the family planning but will be impregnating her, it’s not the best. When I spoke to the guy, he got angry on the phone and told me that he is the one taking care of the lady so he has the full right so the lady shouldn’t come home with any family planning method and that he knows all about our commodities so if we do anything for the lady he the man will know. The lady couldn’t go home, she was here crying because according to her that wasn’t the first termination………Midwife, General Hospital*

The quote above highlights the powerlessness of some women in non-marital sexual relations. Some male partners almost exercise full control over spouses.

### Special circumstances: Teenagers and covert behaviours

Nurses expressed the view that while teenagers were the least likely to be consumers of FP amongst sexually active girls and women, teenage girls perhaps had the highest need for contraceptive concealment as they were not expected to be having sex and may not have the resources to access any of the covert strategies.

For example, they may not have mobile phones to have discrete conversations with nurses, or may not be able to travel the distance to access FP outside of their immediate neighbourhood. Further, teenagers have very little exposure to the health care centres and hence have little contact with nurses to be counselled on the right course of action to meet their reproductive health needs. These factors limit teenagers’ ability to access FP especially within a community setting where people know a lot about each other.

Despite these multiple level barriers, teenagers and health providers still find a way to improve contraception access at facilities. Providers are encouraged to identify and counsel teenagers who have contact with the clinics:

*This is also important because teenage pregnancy is becoming too much and we have been told to help them so anytime we come into contact with them, we give them counselling to make the right choice. Especially those who come here with unwanted pregnancies they’ve terminated half way. When they come the doctors help them and send them to us to counsel them to choose the right method……… Midwife, Quasi Government Hospital*

In large health facilities, adolescent corners are isolated from the general outpatient departments. Adolescent corners therefore provide the space/privacy and the professional competence to manage reproductive health amongst that age group. In smaller community clinics however where spacing is limited, nurses have to find a way to address reproductive health challenges with a lot of tact and skill especially in the case of adolescents. At the same time, some providers felt that the fears associated with encountering health facilities came from real experiences and were not just misconceptions. A nurse admitted that some health care workers could potentially subject teenagers to some form of abuse.

*At that age especially those in school, they feel when they come here, we might insult them and ask them what they want family planning services for. Especially when they are coming they will be thinking about what people will be thinking about them. That makes a lot of them not come to us and they will be moving to the pharmacy to buy “Secure”. They think when they come the nurse will insult me and what the people around them will say so they will not come. Because she is in school and has not grown yet but she is coming in for family planning… personally I will not insult you or judge you but another colleague can do that …….. Community Health Nurse, CHPS Compound*

As this respondent indicates, not all health care workers share a common support for covert contraceptive health provision. Further, our respondents observed negative attitudes regarding family planning use for some of their patients which can further jeopardize the already limited access to reproductive health in the community.

### FP opportunities

Amidst all the challenges associated with family planning, reproductive health services are delivered with a large degree of success. Nurses understand the complexity and the challenges and work within it. These urban poor women with limited resources still receive almost the full complement of reproductive services provided by the health services. The intervention of nurses make way for women to have all the needed services to improve the circumstances of their lives. These pathways created by healthcare workers ensure that anyone would receive FP services if they can contact the health services at any level.

## Discussion

This study applied qualitative research methods to explore ways in which women conceal contraceptive use from their partners and the role that health care providers have played and strategies they have employed to support CCU. Most of the respondents interviewed worked and lived within the communities where the health facilities were located and had firsthand knowledge of the practices and beliefs about FP in their communities. The multiple level and multiple perspectives of respondents in this study is essential in understanding how the health care system has been managing CCU over the years.

Narratives from the health providers reveal clear patterns concerning covert use. The use of certain FP methods are preferred by clients due to ease of concealment, the time of the day women approached nurses, discrete access and information keeping, non-verbal communication and detailed personal relationships with nurses. These were methods deployed by health workers to help women receive contraception covertly. At the same time, the detailed procedures that providers had to use to support their clients also reveal major community-level barriers to free and open access to and use of FP, particularly for teenagers, as well as serious consequences for revealing clients’ use of FP.

For most studies, women were often the units of observation and units of analysis. These studies tended to report CCU from the perspectives of women, which is understandable given that CCU is essentially a woman’s attempt to gain reproductive control of her body. While these studies provide insights into CCU from the perspectives of women, they are scant on the ways in which the health care system had facilitated CCU. This study has provided perspectives on ways that the health system has supported the desire of women to have some level of reproductive autonomy. More than 25 years after International Conference on Population and Development (ICPD) in Cairo, the empowerment of women to make reproductive choices regarding their own bodies remain a distant reality for some women, even in the capital city. As it stands, CCU may perhaps be the only choice for women entrapped in difficult relationships or even in relationships that are heavily influenced by societal expectations. This study also revealed that the attitudes of men were not the only barriers to contraceptive use. Sometimes women were themselves the barriers to contraceptive use even when their health necessitated it. Perspectives from this study could inform the conduct of family planning intervention programs especially in low income areas where women generally have little reproductive autonomy. FP programs need to properly recognize the contribution of CCU and provide the needed support, especially for lower tier health facilities that provide direct community service to underserved women.

The organization of the Ghana Health Service may not have fairly distributed health care resources in terms of the provision of FP services. There were major disparities in FP resources allocated to the different levels of health care provision. In the major regional and district facilities where health facilities had more resources and were quite detached from community members, the provision of family planning services was focused on having all the methods and options available. They did not have regular direct outreach community services. By their own account, they had enough resources to provide services including sufficient and sometimes separate spaces for procedure and counselling. Covert behaviours associated with large facilities were expressed in terms of the choice of method. The contrast was rather striking for smaller community clinics who were provided limited FP resources. Health providers working at CHPS compounds directly encountered the community, where myths, misperceptions and fears played a unique role. Covert behaviours expressed were varied because the facilities were located in the community. Furthermore, community health nurses provided house-to-house care where direct community engagement helped to support FP related activities where it mattered the most. These opportunities notwithstanding, the provision of FP within the community was complicated, and this explains why detailed procedures were necessitated for the provision of covert FP. Health providers working within the context of CHPS were therefore more exposed to threats associated with the provision of FP within the community. They also regularly encountered the consequences of not using contraceptives. In recognition of the mismatch in resources between higher level facilities and lower tier facilities, the GHS has developed elaborate referral systems that rarely work in reality. As nurses admit, many community members are not willing and able to commit extra resources to travel the distance and navigate complex hospital procedures when there are seemingly easier unregulated alternatives in the community.

Scholarly discussion on CCU in urban Accra is complex, particularly in the context of Accra where Ga women are the dominant ethnic group and also because it is the capital. Accra women, over the years, have received a lot of scholarly attention especially from anthropologists as a result of the unique residential arrangement of the Ga marriage [23–25]. Traditionally, Ga wives and husbands did not share the same marital home; wives continued to live in their father’s house while the man also lived in his own house. A wife only came to her husband’s house to fulfill her sexual obligation and returned to her father’s house that same day. This duolocal family arrangement limited the control men exerted over their women and promoted some level of autonomy. In addition, the presence of the capital in Accra promoted commerce; women’s access to their own income due to trade in Accra further empowered them relative to women in other areas of the country. There is evidence to show that Ga women have dominated the fish trade in Accra for generations [26]. It is however not immediately clear whether financial and other kinds of autonomy translate into reproductive autonomy. Recent evidence shows that despite the relatively high level of financial autonomy historically associated with Ga women, their sexuality is still subject to their spouses even though some researchers disagree [6, 27]. Evidence from this study suggests that women’s sexuality may have multiple level influences and individual level studies alone may not sufficiently explain the sexual autonomy associated with the Ga woman. For example, evidence from this study shows that besides partners, other women and the community at large exert some kind of influence over the desire to use contraceptives. This study lends support to the notion that financial autonomy may not directly translate into sexual autonomy. This explains why FP decisions and use are subject to covert practices because of the power imbalances within the context of unions.

In an era of universal health coverage, there is a real opportunity to improve access and use of contraceptives. Although the climate of health policies in Ghana favours access and use of contraceptives, this is challenged by strong patriarchy where women have limited autonomy. Health policies need to both support the open use of contraceptives and also respond equally to more than 30% of women who use contraceptives covertly in Ghana [3]. Policies need to make provision for the protection of women and health care providers from aggrieved partners during episodes of disclosure. Finally, the health system needs to properly acknowledge covert use by maintaining routine records on CCU to better understand the phenomenon in Ghana and adjust policies and programs accordingly.

## Conclusion

This study identifies five key ways that health facilities assist women to conceal contraceptive use from the partners. These are (a) easily concealed-methods, (b) discrete-access-and-information-keeping, (c) time-of-day, (d) non-verbal-communication and (e) use of relationships. The fact that the health institutions were prepared to sometimes perform complex manoeuvres highlights the social weight of contraceptive use amongst community members in many developing countries. Studies like this might help explain the persistently poor contraceptive prevalence rates in many African countries. Furthermore, the unmet need for contraceptive use may perhaps be reduced by improving access to contraceptives discretely. Accepting the social weight of contraceptive use, observing the dimensions of it and designing programs that are sensitive to the nuanced community representation of contraceptive use may perhaps advance the impact of programs better than then the denial of it.

## Data Availability

Dataset will be made available by the corresponding author upon reasonable request.

## Abbreviations

CHPS: Community Based Health Planning and Services
CCU: Covert Contraceptive Use
DDNS: District Director of Nursing Services
FP: Family planning
GHS-ERC: Ghana Health Service Ethical Review Committee
UG-ECH: University of Ghana Ethics Committee for the Humanities
